# Nanometer-Sized Boron Loaded Liposomes Containing Fe_3_O_4_ Magnetic Nanoparticles and Tributyl Borate and Anti-Albumin from Bovine Serum Antibody for Thermal Neutron Detection

**DOI:** 10.3390/ma14113040

**Published:** 2021-06-03

**Authors:** Wei Zhang, Kaikai Wang, Xiaodan Hu, Xiaohong Zhang, Shuquan Chang, Haiqian Zhang

**Affiliations:** 1 College of Materials Science and Technology, Nanjing University of Aeronautics and Astronautics, Nanjing 211100, China; zhangwei_nuaa@outlook.com (W.Z.); wkk_nuaa@163.com (K.W.); huxiaodan@nuaa.edu.cn (X.H.); zhangxiaohong@nuaa.edu.cn (X.Z.); 2 Jiangsu Key Laboratory for Biomaterials and Devices, Southeast University, Nanjing 210096, China

**Keywords:** neutron detector, magnetic liposome, methylene blue (MB), anti-BSA, surface plasmon resonance

## Abstract

A shortage in the supply of ^3^He used for thermal neutron detector makes researchers to find ^3^He alternatives for developing new neutron detectors. Here, we prepared a neutron-sensitive composite liposome with tributyl borate and encapsulating with Fe_3_O_4_@oleic acid nanoparticles (Fe_3_O_4_@OA NPs), methylene blue (MB), or anti-albumin from bovine serum (anti-BSA). The tributyl borate compound was characterized by Fourier transform infrared spectroscopy (FT-IR). In addition, the morphology, element compositions, and magnetic properties of the composite liposome were investigated with transmission electron microscopy (TEM), energy-dispersive X-ray spectroscopy (EDS), and vibrating sample magnetometer (VSM), respectively. The results indicated that a typical ellipsoidal magnetic liposome structure was obtained, and the lengths of the minor axis and major axis were 49 ± 1 nm and 87 ± 3 nm, respectively. Under thermal neutron irradiation, the structure of composite liposome was destroyed, and encapsulated reporter molecules were released, which was detected by ultraviolet–visible (UV–vis) spectroscopy and surface plasmon resonance (SPR) technology. The response of this sensor based on a destructive assay shows a good correlation with neutron doses. Besides, the sensor has a neutron to gamma-ray rejection ratio of 1568 at a thermal neutron flux rate of 135.6 n/cm^2^·s, which makes it a promising alternative to ^3^He.

## 1. Introduction

Neutron detectors are considerably significant for homeland security and civilian application, since neutrons are specifically used for monitoring fissile materials, including those for nuclear weapons in nuclear terrorism. ^3^He-filled gas counters are generally considered as the gold standard for neutron detection. However, the supply of ^3^He, mainly originated from the decay of tritium, is in a shortage due to the ceased production of tritium used in the nuclear weapons programs in the U.S. (Air Products and Chemicals, Hansford Country, TX, USA) and Russia (Gazprom, Moscow, Russia) [1,2]. To address the issue, a variety of ^3^He alternatives have been studied and developed for neutron detectors. Initially, some materials with high nuclear reaction cross-sections such as lithium-6 (Li-6) and boron-10 (B-10) were used as the neutron conversion layer. Britvich [3] manufactured a boron-containing molded plastic scintillator to detect fast neutron. Ryzhikov [4] used the block polymerization method of polystyrene with luminescent dopants and allydodecaborane to obtain a plastic scintillator containing boron, which has a diameter of 25 mm and a height of 25 mm, leading to a significant increase in thermal neutron detection efficiency. However, it is limited by the nature of scintillator such as the luminescence properties. As a result, semiconductor neutron detectors had been focused.

Some semiconductor materials doped/combined with neutron-sensitive materials have also been introduced for the neutron detection and shown better detection performances. At the beginning, the neutron semiconductor detectors were made by coating silicon diodes with boron [5]. Subsequently, with the development of boron nitride (BN) semiconductor materials, some new progress has been made, from simple BN semiconductor layer [6] to the use of hexagonal BN (h-BN) [7], palisade structure design [8], and subsequent applications of pure h-BN as a whole reaction material [9]. All these improve the detection efficiency of the radiation sensor. However, a key point in the design of semiconductor detector with multilayer structure lies in the optimization of sensitive layer thickness, which requires a compromise between increasing the probability of nuclear reactions and effectively separating carriers. To avoid these thorny problems, some new approaches have emerged.

Nanoparticles often have some unexpected benefits and has been attempted to improve the flexibility of radiation detection, which allows it to be applied in many occasions. For instance, Zheng [10] reported a direct conversion solid-state neutron detector by combining the B-10 with sub-2 nm Pt nanoparticles. Koirala [11] placed B-10 nano/micro particles in channels of silicon semiconductors by electrophoretic deposition to detect thermal neutrons. Amaro [12] proposed a proportional counter filled with B-10 nanoparticle aerosol for neutron detection. In general, nanoparticles are more functional than semiconductor materials and can be designed with more specific goals.

It is worthwhile to note that a few of new detection methods and principles are also employed for neutron detection by measuring the change in the structure or concentration of the materials containing sensitive elements to the neutrons. For example, Sun [13] used a surface enhancement Raman scattering (SERS) method for neutron detection, in combination with 4-mercaptophenylboronic acid (4-MPBA) as a detection material. The reaction of ^10^B isotope in 4-MPBA with thermal neutrons converted the molecules into thiophenol, resulting in the changes in SERS spectra of the two molecules adsorbed on a substrate of gold nanoshells. The detection limits of the SERS method were 5.6 μGy and 33.6 μGy for the 4-MPBA adsorbed on solid phase substrates and dissolved in solvent, respectively [13].

Liposome is a cellular mimic with artificial membranes, structurally similar to the lipid bilayer and cytomembrane [14,15]. The membrane structure possesses the advantages of convenient surface modification and being functionalized with molecules and biomolecular groups, such as proteins [16,17]. Importantly, these functionalized liposomes have been extensively employed in the development of novel sensors [18,19,20,21]. Liposomes have usually been used as carriers for signal molecules, which could be released upon interacting with analytes, and detected by using appropriate detection methods [20,21]. By modification of the biorecognition molecules, e.g., biotins used in competitive immunoassays, onto the liposome surface, versatile substances can be targeted and thus further quantified. Electrochemical sensor-based methods are favorable due to their simplicity and ease of operation. However, the sensitivity of electrochemical detection is limited, owing to diluted signal molecules released from liposomes into the surrounding medium. To address this issue, magnetic beads are used to enrich signal molecules on the electrode. The approach, however, is complicated and increases the cost of analysis [22,23].

In addition, there have been significant studies on boron-containing drugs used in boron neutron capture therapy (BNCT), thus promoting research on new materials for neutron detection. Hiroyuki Nakamura [24,25,26,27] synthesized a cage-like double-tailed boron cluster lipids B_12_H_11_SH (BSH), in which the vertices were boron atoms. The long-chain amphipathic molecules of BSH were set in the double lipidic layer (DLL) of liposomes. Accordingly, this greatly increased the loading of boron and the probability of neutron reaction in the liposomes.

Surface plasmon resonance (SPR) is a kind of physical phenomenon at an interface of metal with a dielectric medium, and has become a highly sensitive technique used in sensors and biosensors. Localized SPR (LSPR) technology can be used to enhance scintillators. Bingell [28] synthesized core–shell nanoparticles for use in liquid scintillators, due to the LSPR effect, the synthesized Ag@SiO_2_ nanoparticles could enhance the light yield of scintillator. Liu [29] demonstrated that periodical arrays of Ag nanoparticles can enhance the light emission from a plastic scintillator layer on the surface of a silicon substrate. The enhancement is attributed to surface lattice resonances with a photonic-plasmonic nature. 

In general, the combination of SPR and radiation detection requires a proper design, and SPR is most used for molecular detection. A variety of works have been reported for the molecular and biomolecular detection based on SPR technology [30,31,32,33,34]. We also reported a SPR-based bio dosimeter in combination with hypoxanthine phosphoribosyl transferase (HPRT) genes by detection of the HPRT concentration in the serum of an irradiated mouse [35]. In our earlier work [36], we successfully constructed the highly sensitive gold nanoparticles-DNA nanosensor for γ-radiation detection, and SPR significantly improved the detection sensitivity.

The aforementioned works inspired us to introduce ^10^B-containing molecules, synthesized with a variety of ways [37,38], into the structure of the liposomes for developing new neutron-sensitive materials to address the issue of ^3^He shortage. 

In this paper, a boron-containing liposome sensor was designed and prepared for neutron detection. A boron-containing amphipathic compound was prepared, and then incorporated into the liposome structure. In addition, Fe_3_O_4_ nanoparticles were encapsulated in the liposome for magnetic separation, and MB and anti-BSA were used as reporter molecules. The liposome structure was disrupted under neutron irradiation because of the reaction of ^10^B with neutrons, resulting in the release of the encapsulated markers, which were separated from the intact liposomes by magnetic separation. The neutron irradiation could be detected by measuring the concentration of released markers. To our knowledge, this is the first time that the structure of a liposome was used to detect neutrons, providing a new strategy for neutron detection. 

## 2. Materials and Methods

### 2.1. Chemicals

The reactant of esterification n-butyl alcohol (CAS NO. 71-36-3), boric acid (CAS NO. 10043-35-3), and the solvent toluene (CAS NO. 108-88-3); and other chemicals used in the experiment like methylene blue (MB), chloroform (CAS NO. 67-66-3), and methyl alcohol (CAS NO. 67-56-1) were purchased from Aladdin reagent (Shanghai, China). The cholesterol and phospholipids for preparation of liposomes were purchased from Macklin (Shanghai, China). Antialbumin from bovine serum (anti-BSA) was purchased from Bioss Antibodies reagent (Bioss, China). Fe_3_O_4_ coated by oil acid nanoparticles (Fe_3_O_4_@OA NPs) were kindly provided by Southeast University, China. The water used for the experimental purposes was ultrapure water with a resistivity of 18.25 MΩ·cm, obtained by a purification system (EPED-E2-10TF, Nanjing Yipu Yida Development Co., Ltd, Nanjing, China). 

### 2.2. Synthesis of Tributyl Borate

**Figure 1:**
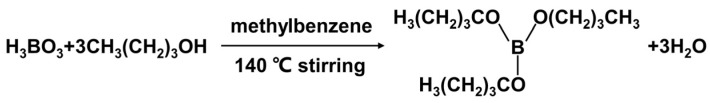
Esterification reaction for boron-containing amphipathic molecule.

The compound molecules containing boron were synthesized by esterification reaction [39] as shown in Figure 1. In a typical reaction, 8 g (0.13 mol) boric acid, 37.68 mL (0.40 mol) n-butyl alcohol, and 87.06 mL (0.82 mol) methylbenzene were added to a 250 mL three-neck round-bottom flask with an oil–water separator, thermometer, and magnetic stirrer (the device diagram was shown in Figure S1 in supplementary materials). The reaction was kept at 140 °C for 3 h. The solvent methylbenzene was evaporated under vacuum. Finally, the product (tributyl borate) was dried under vacuum and frozen.

### 2.3. Preparation of Magnetic Liposomes Containing Boron and MB and Anti-BSA

The liposome was prepared by a reverse-phase evaporation method (RPEM) using ultrasonication. Briefly, phospholipid (0.5 g), cholesterol (0.25 g), and Fe_3_O_4_@OA nanoparticles (NPs) (0.1 g) were dissolved in 30 mL oil phase, which was mixed by chloroform and methyl alcohol (2:1 *v*/*v*); tributyl borate (0.5 g) and MB (0.2 g) were dissolved in 15 mL water phase (water: methyl alcohol = 1.5:1 *v*/*v*) and dissolved for 30 s. Then, the oil and water phase were blended under ultrasonication. Finally, the obtained liposomes were separated by magnetic separation, and washed by water and ethyl alcohol for 3 times. The products were stored in water. The same method was used to prepare antibody-containing material, only the added MB was replaced by the anti-BSA antibody (0.02 mg). For the control group (MB-containing without boron), all the experimental procedures were consistent with those before, except that tributyl borate was not added. 

### 2.4. Characterization of Materials

FT-IR was used to characterize the tributyl borate. The morphologies and elemental compositions of liposomes were observed by transmission electron microscopy (TEM) and energy dispersive spectrometer (EDS). The thermostability of Fe_3_O_4_@OA NPs and composite liposomes was studied by thermogravimetric analysis (TGA) measurement. The magnetic properties of liposomes were measured by a vibrating sample magnetometer. All details could be found in the Section 2 of supplementary materials.

The influence of different ultrasonic conditions such as temperature (35, 50, and 70 °C), power (90, 120, and 150 W), and time (10, 20, 30, and 40 min) on the liposome yield was studied by the method of single control variable. To obtain the yield, the as-prepared liposomes were freeze-dried into a solid state and then the mass (*m*_1_) was recorded. The yield was calculated by Equation (1), where *m*_2_ was the total mass of all solid raw materials for preparing liposomes.

(1) $$yield=\frac{m_{1}}{m_{2}}\times 100\%$$

### 2.5. Neutron Irradiation Experiments

The liposomes were irradiated by an ^241^Am-Be source (300 mCi) with the moderation material (hard paraffin). The irradiation time was set to 0, 0.5, 1, 2, and 8 h, and the liposomes without loading boron was also irradiated as a control. In order to obtain more thermal neutrons, the thickness of paraffin was optimized by utilizing the Monte Carlo N-Particle Transport Code System (MCNP, version 4C). The neutron flux rate at optimal moderator thickness was determined by both MCNP calculation and activation method, and the experimental details were in the Section 4 of supplementary materials. 

### 2.6. Thermal Neutron Detection Performance Analysis

After different irradiation time under the neutron field, the released MB and anti-BSA were collected by magnetic separation. The concentration of MB were measured by UV–vis spectroscopy (UV-2550, Shimadzu, Japan), and the scanning range is from 200 to 900 nm, and the scanning interval is 1 nm. The concentration of anti-BSA was obtained by measuring the binding of anti-BSA and BSA modified on the gold film of a SPR sensor (BI-2000, Biosensing Instrument Inc., Tempe, AZ, USA), the detailed procedures could be found in our previous publication [35]. 

## 3. Results

### 3.1. Characterization of Tributyl Borate

**Figure 2:**
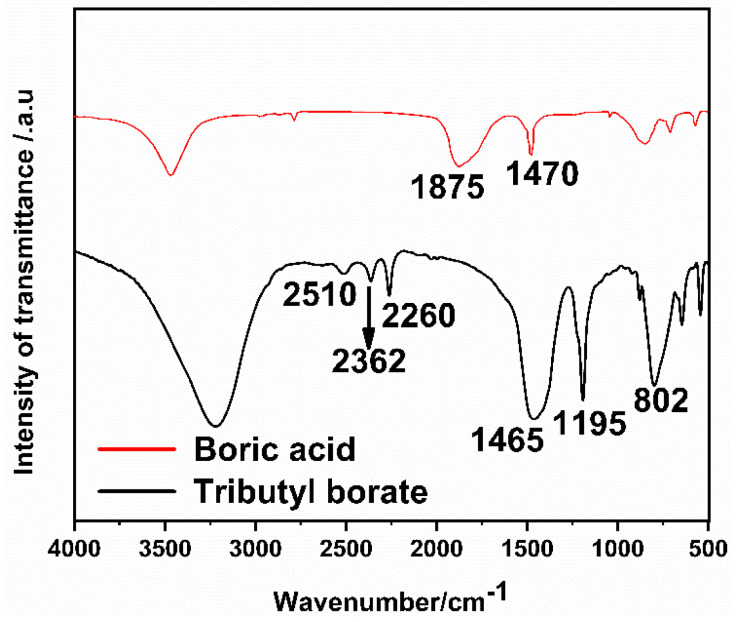
FT-IR spectra of tributyl borate.

As shown in Figure 2, 2510 cm^−1^ of the -C-H vibration absorption peak demonstrated the existence of the methyl, while absorptions at 2362 cm^−1^ and 2260 cm^−1^ are ascribed to -C-H-vibration of methylene of the tributyl borate. These are due to the contribution of the methyl and ethyl groups in n-butanol. By comparing the peak of boric acid (in red) 1470 cm^−1^, it can be judged that it is the symmetric stretching vibration peak of -B-O-, and the 1875 cm^−1^ peak of boric acid is thought to be the vibration peak of -OH on B, but it disappears on tributyl borate (in black), while, the characteristic peak of boric acid ester appears at 1195 cm^−1^ of tributyl borate, which is consistent with the fact that borate ester takes off the hydroxyl group and forms borate ester bond [39]. The peak at 802 cm^−1^ could be originated from absorption of B-O symmetric stretching vibration peak. These results indicated that tributyl borate was successfully synthesized.

### 3.2. Process of Magnetic Liposomes Preparation

**Figure 3:**
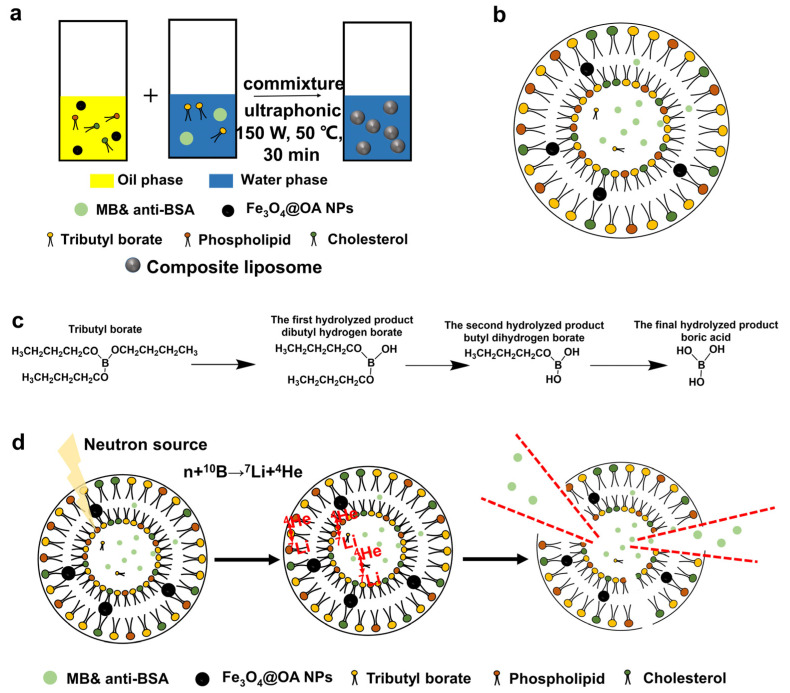
Process of the preparation of composite liposome by RPEM (**a**), diagrammatic cross-section of composite liposome (**b**), hydrolytic process of tributyl borate (**c**), and schematic diagram of the material released after neutron irradiation (**d**).

Figure 3a shows the preparation process, and Figure 3b was the schematic diagram of the as-prepared liposomes. Both the external continuous phase and internal phase of the liposome structure are water, while the hydrophobic structure of liposomes is in the middle of the hydrophilic layer, forming the special structure of liposomes with an ellipsoidal shape. Certainly, phospholipid and cholesterol constitute the DLL structure of liposomes and Fe_3_O_4_ NPs modified by oleic acid exist in liposomes’ hydrophobic layer. MB can be dissolved in both water and organic phase, while anti-BSA exists only in the internal water phase. The synthetic tributyl borate also contributes to the structure of liposomes. Since borate ester bond is easy to hydrolyze, as shown in Figure 3c, it is not possible to accurately predict where the tributyl borate in the liposome. The hydrolysis of tributyl borate in the first two steps produced a hydrophilic hydroxyl group and retained the hydrophobic butyl bond, which facilitated the formation of the DLL structure of the amphiphilic liposome. Boric acid, resulted from complete hydrolysis of tributyl borate, can also be encapsulated in liposomes. In this way, boron can be introduced into liposomes.

In fact, no matter which structure tributyl borate is in the liposome, the reaction between neutron and boron will not be affected, as shown in Figure 3d, hydrolysis process was occurred accompanied with the preparation of liposomes; different hydrolysis products were obtained through the step-by-step hydrolysis, and then they entered different positions of liposomes and different structures of liposomes were formed therefore. In this way, boron can be introduced into different positions of liposomes, to ensure that the liposome structure can be broken when neutron interacts with boron.

### 3.3. Ultrasonic Preparation of Magnetic Liposomes Containing Boron

**Figure 4:**
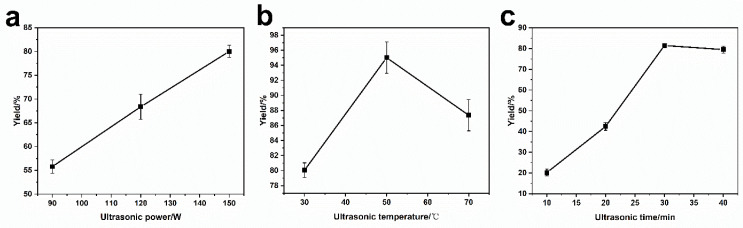
Ultrasonic conditions of liposomes preparation with respect to ultrasonic power (**a**), ultrasonic temperature (**b**), and ultrasonic time (**c**).

From Figure 4a, the yield increased with the ultrasonic power, which could be ascribed to an increase in organic solvent evaporation rate under high ultrasonic power, leading to accelerated the products exchange between organic phase and water phase during formation of the liposomes. In particular, the yield reached the maximum when the temperature was 50 °C, as shown in Figure 4b. Before 50 °C, the increasing temperature promoted the evaporation rate of the organic phase, accelerating the formation of the liposome. In contrast, as the temperature was over 50 °C, water began to evaporate, leading to adhesion of liposomes onto tube wall and a decline in the yield consequently. With the increase of ultrasonic time (Figure 4c), the yield of the liposome increased gradually, and reached a maximum in 30 min. The yield would stay unchanged with a further increase in the reaction time. This is because, with the increase of ultrasonic time, the organic phase was evaporated completely within 30 min, and the liposomes were fully formed. Therefore, preparation of liposomes with amphiphilic molecules containing ^10^B, phospholipid, cholesterol, and MB were realized in a maximum yield under ultrasonic power 150 W for 30 min at 50 °C.

### 3.4. Characterization of Magnetic Liposomes

**Figure 5:**
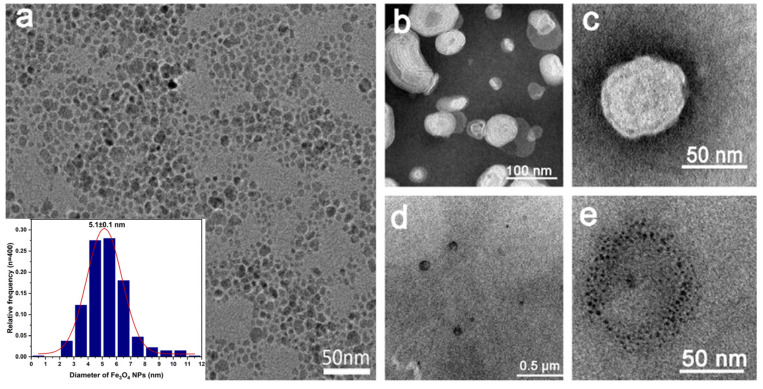
Characterization of Fe_3_O_4_@OA and composite liposomes. (**a**) TEM images of Fe_3_O_4_@OA and Inset: Size distribution of Fe_3_O_4_@OA (average diameter = 5.1 ± 0.1 nm). (**b**,**c**) Liposome@ Fe_3_O_4_ with negative staining. (**d**,**e**) Liposome@Fe_3_O_4_ without negative staining.

Figure 5a–e exhibits the TEM images of the Fe_3_O_4_@OA NPs and the composite liposomes. Figure 5a indicates that the Fe_3_O_4_@OA NPs were with good dispersion and uniform size, and the average particle size was approximately 5.1 nm with a standard deviation of 0.1 nm. Figure 5b–e shows the structure of the composite liposomes. To visualize the structure of liposomes, negative staining with the phosphotungstic acid technique was used. After being negative stained, the ellipsoidal composite liposomes were clearly visible. The lengths of the minor axis and major axis of ellipsoids were 49 ± 1 nm and 87 ± 3 nm, respectively. Furthermore, the flattening of ellipsoid was calculated to 0.44 ± 0.04 according to the error propagation formula (details were in the Section 3 of supplementary materials). Figure 5d,e are the TEM images for the composite liposomes without negative staining treatment. Many black ellipsoid materials can be seen in Figure 5d, which is approximately the same size as composite liposome. A magnified image is shown in Figure 5e, which shows that the ellipsoid was made up of smaller dispersed Fe_3_O_4_@OA NPs, which form a ring. The Fe_3_O_4_@OA NPs were modified with oleic acid, leading to preferable distribution of the encapsulated nanoparticles in the proximity to the oily ring structure of the liposomes. The size of the annular structure formed by the encapsulated nanoparticles matches the size of the liposomes. Summarily, these results demonstrated successful preparation of the designed liposomes.

An EDS measurement was performed to characterize the ratio of the elements of Fe_3_O_4_@OA NPs and liposomes, the results are given in Table 1 and Table 2. The distribution of elements Fe, C, H, and O in Table 1 was consistent with the composition of the Fe_3_O_4_@OA NPs. In Table 2, boron and phosphorus were derived from liposome structures; sulfur and chlorine are from methylene blue. The value of B/P was 3.5, which is high enough to meet the requirement of BNCT [25,26,40]. The boron content of the composite material was sufficient for nuclear reaction with neutrons.

Comparing the two groups of element analysis, it can be clearly seen that the boron was successfully introduced into the structure of liposomes. Besides, the probability of the reaction between boron and neutron is independent of where the boron is located in liposomes. It provides a theoretical and practical basis for the subsequent experiments. Similarly, the calculated B/P ratio shows that the boron content was sufficient to meet the requirement of destroying the liposome structure. In addition, the existence of Fe element can ensure the requirement of magnetic separation and facilitate subsequent detection, which is consistent with the separation experiment results.

**Figure 6:**
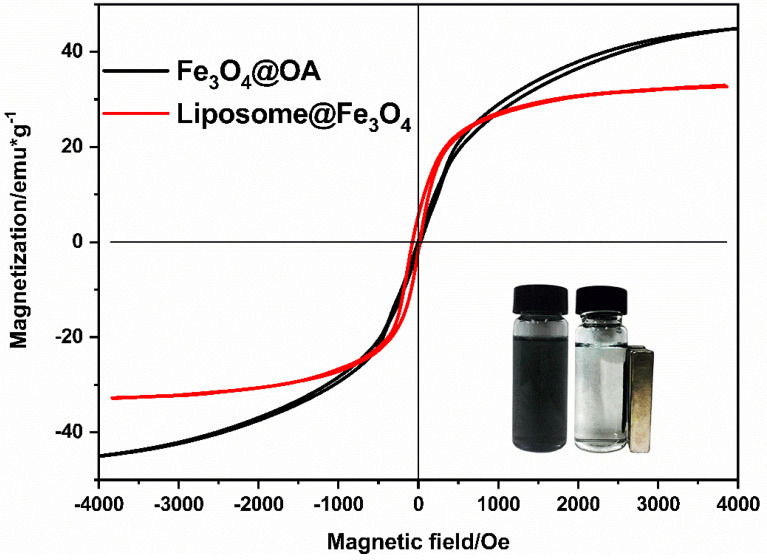
Magnetization curves of Fe_3_O_4_@OA nanoparticles and liposomes@Fe_3_O_4._

The magnetization curves for Fe_3_O_4_@OA and Fe_3_O_4_-encapsulated liposomes (liposome@Fe_3_O_4_) are shown in Figure 6. The figure presents typical plots of magnetization versus the applied magnetic field, and the appearance of the hysteresis loop indicates that both the Fe_3_O_4_@OA and liposome@Fe_3_O_4_ possess good paramagnetic properties. The saturation magnetization value was measured to be 45.184 emu/g for Fe_3_O_4_@OA and 31 emu/g for liposome@Fe_3_O_4_. The loss of magnetization is due to the presence of liposomes. The coercivity, remnant magnetization, saturation magnetization of liposome@Fe_3_O_4_ are 14 Oe, 5.8 emu/g, and 31 emu/g, respectively. These results indicate that the liposomes show paramagnetism due to the Fe_3_O_4_@OA encapsulation. Although the saturation magnetization decreased in the liposome@Fe_3_O_4_, the complete magnetic separation of liposome@Fe_3_O_4_ samples were still achieved in 2 min with a magnet, which was put near the tubes containing the aqueous dispersion of the liposomes, as shown in the inset of Figure 6. After magnetic separation, the UV–vis detection of MB will not be interfered by the liposome composite materials. The value of MB concentration is much more believable. Fe_3_O_4_@OA was used of its role of magnetic adsorption, to achieve the purpose of magnetic separation. The products after irradiation contain released MB and Fe_3_O_4_@OA, damaged liposome fragments structure, and intact liposomes. Under the magnetic field, the non-damaged liposome structure and the released Fe_3_O_4_@OA can be separated rapidly, and the released MB and the liposome fragments are therefore obtained. Furthermore, the UV-vis absorption is characteristic, so the change of MB concentration can be clearly studied. Similarly, the addition of antibodies improves the accuracy of the detection greatly, because the antibody–antigen binding is unique.

**Figure 7:**
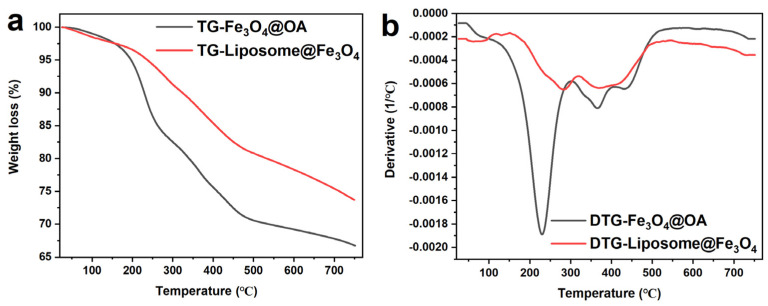
TG (**a**) and DTG (**b**) of Fe_3_O_4_@OA NPs and liposome@Fe_3_O_4._

The thermal stability of the composite liposome and Fe_3_O_4_@OA NPs were studied by synchronized thermogravimetric (TG, Figure 7a) and derivative of TG (DTG, Figure 7b). The TG graph shows that three predictable weight losses have been found of Fe_3_O_4_@OA NPs, and the primary weight loss is occurring at a temperature before 260 °C owing to desorption of oil acid; the second weight loss is occurring in the range from 260 to 480 °C due to of decomposition of unrefined content; the final weight loss is observed between 256 and 470 °C due to crystallization turning medium. Similarly, there were four predictable weight losses of liposomes in the Figure 7a. The primary weight loss is occurring at a temperature before 150 °C owing to desorption of liposome component, and the following weight losses was similar to the process of Fe_3_O_4_@OA NPs. In general, the DTG curve indicates an exothermic and endothermic reaction of the as-prepared materials, in which the DTA curve showed that two endothermic peaks occurring at 321 °C owing to dehydration and at 460 °C owing to decomposition of anhydrous precursor are obtained. The exothermic peak at 530 °C reveals the phase transformation of Fe_3_O_4_. In general, the liposome structure is easily removed by heat, but is sufficient for use at room temperature.

### 3.5. The Measurement of Thermal Neutron

**Figure 8:**
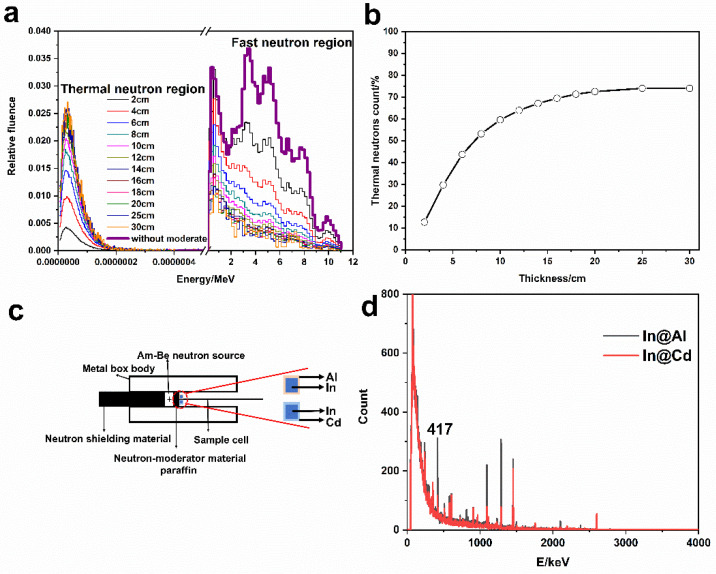
Neutron energy spectrum at different thicknesses of moderator (**a**), proportion of thermal neutrons in total neutrons (**b**), diagram of experimental apparatus for measuring neutron flux rate by activation method (**c**), and gamma spectrum of activated In sheet covered by Al and Gd, respectively (**d**).

In MCNP, the neutron energy spectrum and percentage of thermal neutrons under different thicknesses of paraffin were shown in Figure 8a,b. Overall, a high proportion of thermal neutrons is expected. When the thickness was less than 20 cm, the proportion of thermal neutron progressively increased and then it reached the saturation (72.5%). Further, the thermal neutron flux rate was calculated to 127.4 neutrons/cm^2^·s. As a consequence, 20 cm was as the optimal thickness of the moderator. On the other hand, the activation experiments indicated that in was activated and the gamma ray energy spectrum was measured (Figure 8d), and the counts at 417 keV were focused. In view of neutron self-shielding effect, the neutron flux rate experimentally was 135.6 neutrons/cm^2^·s. The details could be found in the Section 4 of supplementary materials. The relative error between this experimental value with the theoretical calculation was only 0.66%. Meanwhile, the absorbed dose rate of gamma ray under the neutron field was measured to 3.91 μSv/h by a gamma dosimeter (FD-3013H, Shenghe, Shanghai, China). 

### 3.6. Release of MB and Anti-BSA Loaded Liposomes 

**Figure 9:**
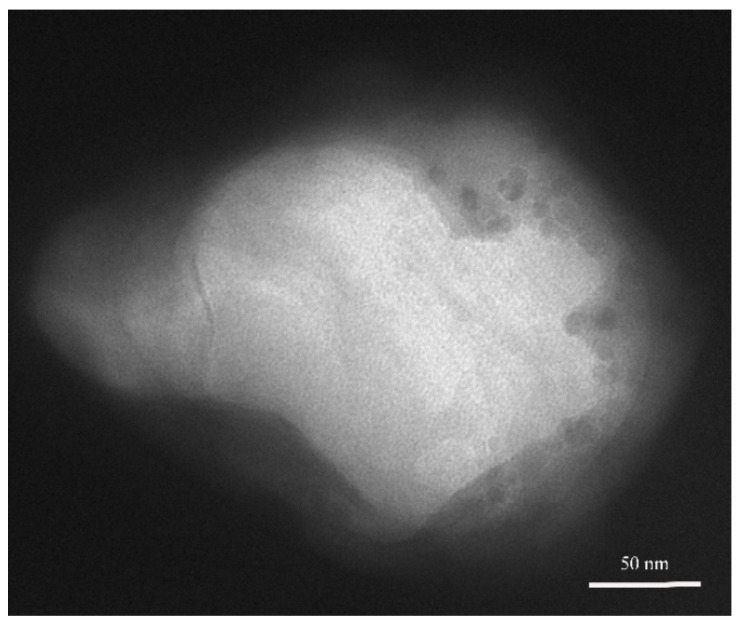
TEM image of liposome after neutron irradiation.

It was expected that the structure of liposomes can be destroyed after neutron irradiation, and the encapsulated molecules were released to achieve the purpose of neutron detection. Figure 9 is the TEM image of the irradiated liposome sample. The structure of liposome cannot be maintained at all, presenting a vague morphology. Some Fe_3_O_4_ particles can be observed outside. Compared with Figure 5c, there is no ellipsoidal liposome morphology. What’s more, the size could be larger than unirradiated liposomes. In this way, the structure of liposome was destroyed under the neutron field, which agreed with the expectation.

**Figure 10:**
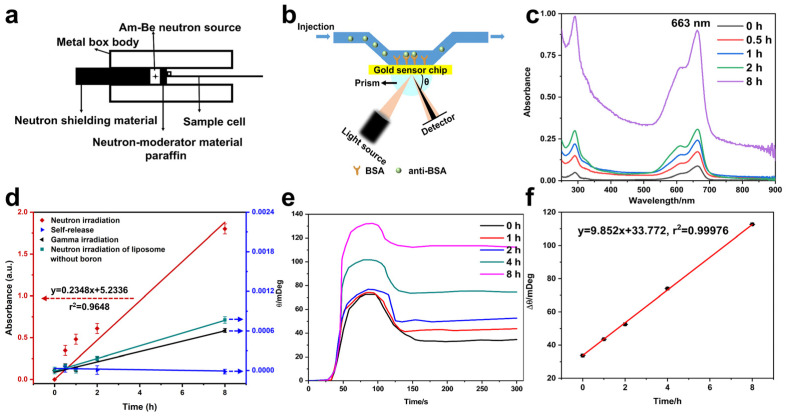
Release detection after irradiation, schematic diagram of radiation device (**a**), schematic diagram of SPR detection (**b**), absorbance peak of released MB from composite liposome by neutron irradiation (the samples have been diluted twice) (**c**), absorbance analysis of different samples after irradiation (**d**), the resonance angle signal detected by SPR of released anti-BSA (**e**), and the linear correlation of anti-BSA resonance with irradiation time (**f**).

The liposome samples were closely behind the paraffin moderator, as shown in Figure 10a. The change of UV absorption spectrum of methylene blue was measured, as shown in Figure 10c. It has an obvious absorption peak at 663 nm, owing to the conjugated structure in MB molecule. Based on the standard curves of MB (Figure S3), the absorbance at 663 nm was chosen as the research object. The absorbances at 663 nm under four conditions such as neutron irradiation, self-release, gamma irradiation by a Cs-137 source and neutron irradiation without loading boron were measured (Figure 10d). It should be noted that the samples were diluted twice in the absorption spectrum measurement in Figure 10c, and then the actual absorbance was converted according to the dilution factor and standard curves of MB, as shown in Figure 10d. Meanwhile, the samples at t = 0 were as the reference in Figure 10d, leading to the differences of the initial absorbances between Figure 10c,d. With the increase of neutron irradiation time, the more MB were released and detected, and the color of released MB solution was also gradually deepening (Figure S4). The absorbance of MB was linearly related to the irradiation time. The change of liposomes by neutron irradiation was the most significant. The change of absorbance of MB in self-release samples was almost little noticeable, which indicated that the structure of liposomes is complete and the stability is good enough. Continuous monitoring for up to 15 days showed that there was still no significant MB release, while the solution remained colorless and transparent (Figure S5). The experimental details were in the Section 5 of supplementary materials. Gamma irradiation can make liposome structure destroyed, but the change of gamma irradiation for 8 h was similar with self-release. Compared with the changes caused by neutron irradiation, it was negligible. Furthermore, for the liposomes without loading boron irradiated by neutron, the change was also small, which indicated that the boron was essential for thermal neutron detection. The ratio of absorbance of liposomes exposed to neutron field and gamma field for 8 h was calculated to 1568. In other words, liposomes are more sensitive to neutrons than gamma rays. The designed liposome structure can be used to detect the thermal neutron.

The accuracy of detection is greatly improved by using SPR, because of the unique combination of anti-BSA and BSA. BSA was binding on gold film (Figure 10b), the combination of anti-BSA and BSA caused a shift in the resonance angle(θ) of SPR. SPR sensorgram at different neutron irradiation time was shown in Figure 10e, and Figure 10f was the linear fitting of θ to irradiation time. With the increase of irradiation time, the combination of anti-BSA and BSA increased. It has an excellent linear relationship of Δθ and neutron irradiation time. Compared with the UV–vis method, the coefficient of linear correlation of SPR detection method is 0.99976, while it was 0.9648 for the UV–vis method, and the detection error was decreased from 0.03 to 0.003. SPR method can improve the accuracy of measurement, and the detection method has advantages.

## 4. Discussion

Thermal neutrons are neutrons with 0.0253 eV energy and obtained by slowing down the fast neutrons emitted by the neutron source. The boron has a high reaction cross section (σ) of thermal neutrons, which causes the high reaction probability. The following was the two processes of the nuclear reaction equation of neutron and boron.

n+B10→L7i(1.0 MeV)+H4e(1.8 MeV)→L7i(0.83 MeV+H4e(1.47 MeV)+γ(0.48 MeV) σ=3840b

However, it should be noted that the reaction with high reaction cross section here is only the reaction between thermal neutrons and boron-10(^10^B). In other words, the two conditions of ^10^B and thermal neutrons must be satisfied, so that the reaction cross section is so large and the reaction probability will increase. Therefore, the direction of neutron detection efforts is to increase the proportion of thermal neutrons and the content of ^10^B. At the same time, the released secondary particles (^7^Li, ^4^He) can react with other substances in the system, and these secondary reactions can also be used to capture information in detectors, such as semiconductor detectors to generate carrier pairs.

Under the neutron irradiation, the reaction of ^10^B in the amphipathic compounds with the thermal neutrons would disrupt the liposomes, resulting in the release of the encapsulated molecules. In addition, the secondary particles ^7^Li and ^4^He produced from the reaction would also destroy the liposomes. The releases from the composite liposomes were detected with spectrophotometer and SPR, showing the responsive relationship with the neutron flux. The least square fits to the data give the responsive relationships of y = 0.02348x + 5.2336, r^2^ = 0.9648; y = 9.852x + 33.772, r^2^ = 0.99976, with UV–vis spectrophotometer and SPR, respectively. With SPR, the detection efficiency was 1.87%. After irradiation of the composite liposomes with neutrons and γ for 8 h, ratio of the optical absorption of the releases was 1568, indicating that the liposomes are much more sensitive to neutron than γ.

As compared with B layer-sandwiched semiconductor detector reported by McGregor D.S. [41], the detection efficiency of the composite liposome with B/P of 3.35 was lower than 4–11.6% of the semiconductor detector. However, in the composite liposomes, ^10^B in natural abundance was used for the preparation of the liposomes. The detection efficiency is expected to reach 16.7% if the liposomes contain ^10^B in the abundance of 90% and the mass fraction of 30%. 

At present, the detection methods are all offline. However, assuming that a closure device is added in the process of neutron irradiation and connected with SPR pipe directly and magnets are placed in both sides of the middle of the connection line, the online detection will be possible. When the liquid flows into the pipeline of SPR sensor, magnetic separation is performed first, and then the online SPR detection can be done.

## 5. Conclusions

In this paper, the system takes advantage of the high nuclear reaction cross-section between thermal neutron and boron-10 and the thermal neutron-sensitive composite liposome enclosing Fe_3_O_4_@OA, MB, and anti-BSA antibody were prepared and characterized. Fe_3_O_4_@OA encapsulated inside the liposome was used for simple magnetic separation and enrichment of the MB and anti-BSA released from the liposome under neutron irradiation. The concentrations of the released molecules were detected with spectrophotometer and SPR, and shown to be correlated to the neutron doses. The released testing target material is more flexible, which can be changed as appropriate to the detection method, and more accurate detection method can improve the detection performance, so that to improve the detection performance, and this new type of neutron detection method has great potential to detect thermal neutron.

## Figures and Tables

**Table 1:** Elements content of Fe_3_O_4_@OA NPs.

Elements	Fe	O	C	H
Wt%	51	15	31	3

**Table 2:** Elements content of composite liposomes.

Elements	B	C	N	O	P	Fe	H	S	Cl
Wt%	14	40	1	23	4	7	9	1	1

## Data Availability

Data sharing is not applicable to this article.

## Supplementary Materials

The following are available online at https://www.mdpi.com/article/10.3390/ma14113040/s1, Figure S1: Device diagram of the tributyl borate synthetic process, Figure S2: (a) TEM image of liposomes (b) Size distribution of minor axis of liposomes (c) Size distribution of major axis of liposomes, Figure S3: Standard curves of MB ultraviolet absorption peak, Figure S4: Optical photograph of MB released by neutron irradiation at different times, Figure S5:Stability of liposome composite after standing for different times 1 h (a), 360 h (b), the UV-vis absorbance of MB during 360 hours, Table S1: The relationship between the formation water and time, Table S2: Parametric record for calculating thermal neutron flux rate by activation method, Table S3. The parameters of self-shielding effect are considered.

## Abbreviations

OA	oleic acid
NPs	nanoparticles
MB	methylene blue
anti-BSA	antialbumin from bovine serum
FT-IR	Fourier transform infrared spectroscopy
TEM	transmission electron microscopy
EDS	energy-dispersive X-ray spectroscopy
VSM	vibrating sample magnetometer
UV-vis	ultraviolet-visible
SPR	surface plasmon resonance
Li-6	lithium-6
B-10	boron-10
BN	boron nitride
h-BN	hexagonal BN
SERS	surface enhancement Raman scattering
4-MPBA	4-mercaptophenylboronic acid
BNCT	boron neutron capture therapy
BSH	B_12_H_11_SH
DLL	double lipidic layer
LSPR	localized SPR
HPRT	hypoxanthine phosphoribosyl transferase
RPEM	reverse-phase evaporation method
TGA	thermogravimetric analysis
MCNP	Monte Carlo N-Particle Transport Code System
TG	thermogravimetric
DTG	derivative of TG
