# Supplementary material for: Nanometer-Sized Boron Loaded Liposomes Containing Fe3O4 Magnetic Nanoparticles and Tributyl Borate and Anti-Albumin from Bovine Serum Antibody for Thermal Neutron Detection
Source: Materials (Basel). 2021 Jun 3;14(11):3040. doi: 10.3390/ma14113040 (PMC8199906; doi:10.3390/ma14113040)
Supplement: Supplementary file 1 [file materials-14-03040-s001.zip › materials-1215512-supplementary.pdf]

materials

MDPI

Supplementary Materials

Nanometer-Sized Boron Loaded Liposomes Containing Fe_3O_4 Magnetic Nanoparticles and Tributyl Borate and anti-Albumin from Bovine Serum Antibody for Thermal Neutron Detection

Wei Zhang[1], Kaikai Wang[1], Xiaodan Hu[1], Xiaohong Zhang[1], Shuquan Chang[1*], Haiqian Zhang[1,2*]

[1] College of Materials Science and Technology, Nanjing University of Aeronautics and Astronautics, Nanjing 211100, China

[2] Jiangsu Key Laboratory for Biomaterials and Devices, Southeast University, Nanjing 210096, China

* Correspondence: Shuquan Chang, Email: chsq@nuaa.edu.cn; Haiqian Zhang, Email: zhanghq@nuaa.edu.cn

1. Water separation during the synthesis of tributyl borate

Fig. S1 was the device diagram of the reaction process. This process separated the water from the reaction, ensuring that the reaction continued to the direction of the product. The reaction process could be monitored by the amount of water production.

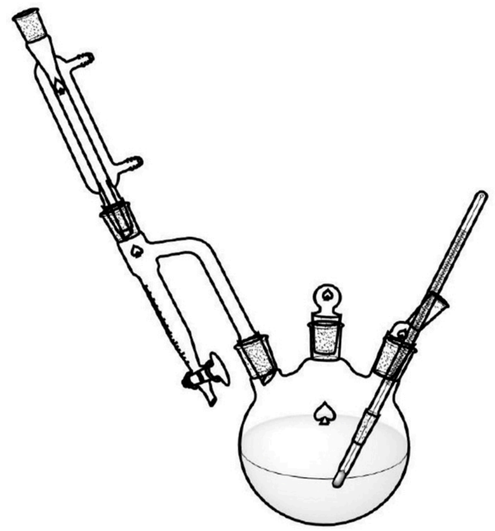

Fig. S1 Device diagram of the tributyl borate synthetic process

2. Details of materials characterization

FT-IR was carried out on the Nexus 670 spectrometer (Thermonicolet, USA) and recorded in the range of 4000 cm^{-1} to 500 cm^{-1}. The voltage of transmission electron microscopy (TEM, JEM-200CX, JEOL, Japan) was 20 kV. The energy dispersive spectrometer (EDS) is acquired by scanning electron microscopy (SEM, JMS-7600F, JEOL, Japan), equipped with Aztec-Xact (Oxford, UK) energy spectrometer, the voltage was 10 kV. Thermogravimetric analysis (TGA, Thermal gravimetric analyzer, Mettler Toledo, Switzerland) measurement was used to study thermo-stability of Fe_3O_4@OA NPs and composite liposome, research temperature was conducted from 35 to 750 °C under argon with heating rate of 20 °C/min and gas flow velocity of 50 mL/min. The magnetic properties were measured by a vibrating sample magnetometer (VSM, VersaLab, Quantum Design, USA) with an applied magnetic field between -4000 Oe and 4000 Oe at room temperature.

3. Determination of size distribution of liposomes

Seventy five liposomes were randomly selected from the Fig. S2(a), and the size distributions of minor axis and major axis of the ellipsoids were statistically analyzed, and they were confirmed to be 49±1 nm and 87±3 nm.

Furthermore, the flattening of ellipsoid (flattening $= \frac{\text{major axis}-\text{minor axis}}{\text{major axis}}$) was calculated to 0.44±0.04 according to the error propagation formula.

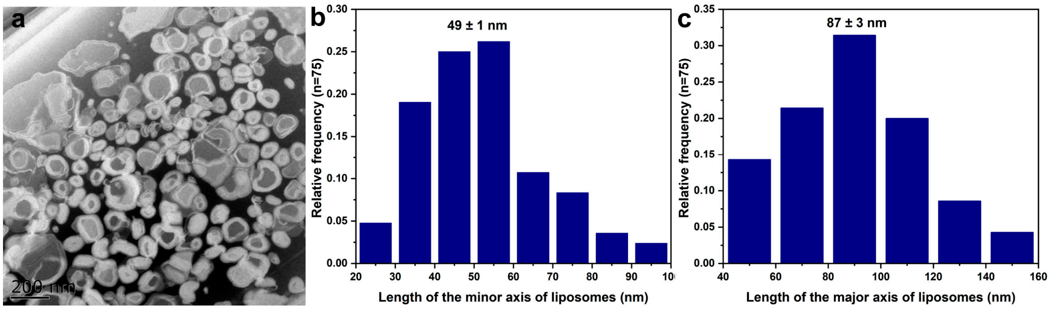

Fig. S2 (a) TEM image of liposomes (b) Size distribution of minor axis of liposomes (c) Size distribution of major axis of liposomes

Table S1. The relationship between the formation water and time

reaction time/min	30	60	90	120	150	180	210
water volume/mL	2.1	3.8	5.4	6.2	6.5	6.7	6.7

Table S1 records the volume of water which indicates the reaction rate during the reaction process. The table shows that the water yield is high in the beginning 120 min, which shows that the reaction speed is very fast at the early 120 min, because of the high concentration of the reactant. After 120 min, the reaction rate became slow, which is due to the lower concentration of reactant and the water was separated, thus the reaction goes in the direction of tri-butyl-phosphate. After 180 min, water yield was smooth and steady, the reaction is over. The reaction time is chosen as 180 min.

4. Calculation of thermal neutron flux rate by activation method

Briefly, the hard paraffin was placed immediately next to the neutron source, and on the other side of paraffin, two pieces of indium (In) with the same size were placed. One was covered completely by cadmium (Cd) to shield thermal neutron, and the other was covered by aluminum (Al) to eliminate the differences in thickness of plates. Activated by thermal neutron, the covered In was picked under the Portable HPGe Gamma Spectrometer (Trans-Spec-N, AMETEK, USA) to obtain the gamma energy spectrum. Formula 1 and 2 was used to calculate the neutron fluence rate after slowing down[1].

$$r = \frac{N_\gamma \lambda A f_s}{MP\eta N_0 I_\gamma \varepsilon_\gamma (1-e^{-\lambda t_0}) e^{-\lambda(t_1-t_0)} (1-e^{-\lambda(t_2-t_1)})} \quad (1)$$

$$\varphi_0 g \sigma_0 = \frac{1}{G_{th}} \left[r_{Al} - r_{Cd} \left(1 + \frac{g\sigma_0}{G_{res} I_0} f_1 + \frac{\sigma_0 w'}{G_{res} I_0} \right) \right] \quad (2)$$

N_γ is the count of characteristic peaks in the gamma spectrum, by integrating over the area; λ is the decay constant; A is the molar mass of In; P is the purity of In in the In sheet; η is the abundance of ^{115}In isotope; M is the mass of In sheet; N_0 is the Avogadro's constant; I_γ is the branching ratio of gamma rays; ε_γ is the detection efficiency of the Portable HPGe Gamma Spectrometer fs is the self-correcting factor[2]; t_0 is the duration of neutron irradiation; t_1 is the time to start measuring; t_2 is the time to end the measurement; $t_1 - t_0$ is the cooling time for In sheet; $t_2 - t_1$ is the measurement time for gamma detection. φ_0 is the thermal neutron flux rate, g is correction factor for thermal neutron energy interval deviating from 1/ V cross section law, σ_0 is the mean cross section of thermal neutron capture reaction, G_{th} is the thermal neutron self-shield factor; r_{Al} and r_{Cd} are experimental value of single nuclear reaction rate of In sheet covered by Al and Cd, Respectively; G_{res} is the the hyperthermal neutron self-shielding factor; I_0 is the resonance integral cross section; f_1 is the correction factor of activation from 5kT to hyperthermal neutrons; w' is the correction factor of activation due to the energy range from 5kT to the range in which neutrons obey the 1/ V rule.

After activation of In, the gamma energy spectrum was measured, and the relevant data was recorded in Table S2 and Table S3. The neutron flux rate could be obtained by putting them into Equations 1 and 2.

Table S2. Parametric record for calculating thermal neutron flux rate by activation method[1]

	In covered by Al	In covered by Cd
N_γ	780.67	170.63
$\lambda(s^{-1})$	2.128×10^{-4}	2.128×10^{-4}
A(g/mol)	115	115
P	0.999	0.999
η	0.957	0.957
m(g)	0.8939	0.9078
N_o	6.02×10^{23}	6.02×10^{23}
I_γ	0.272	0.272
ε_γ	0.0932	0.0932
f_s	1	1
$t_0(s)$	53220	52740
t_1-$t_0(s)$	120	120
t_2-$t_1(s)$	300	300
$\lambda(s^{-1})$	2.128×10^{-4}	2.128×10^{-4}

Table S3. The parameters of self-shielding effect are considered[2]

σ_0	g	$I_0/g\sigma_0$	w'	f_1	G_{th}	G_{res}
166.4±0.6%	1.0194	15.8±0.5	0.2953	0.468	0.744	0.128

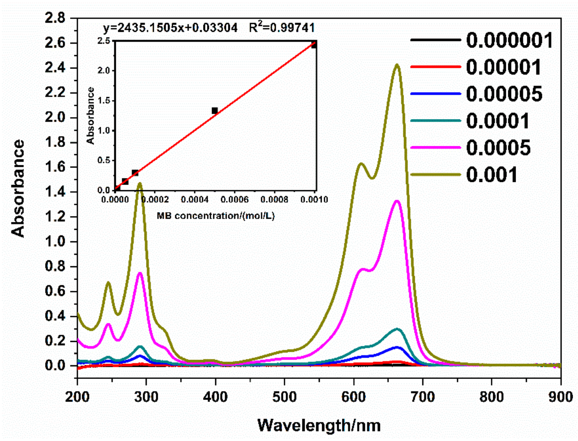

Fig. S3 Standard curves of MB ultraviolet absorption peak

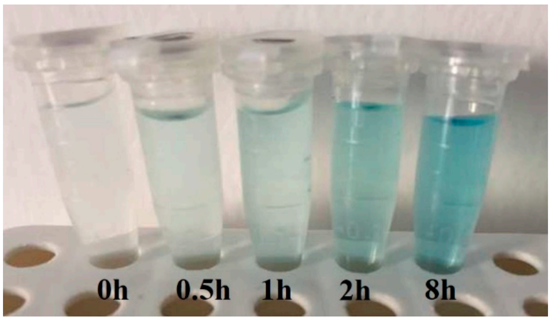

Fig. S4 Optical photograph of MB released by neutron irradiation at different times

5. Stability of liposome composite

In the study of liposomes stability, the freeze-dried liposome powders were dispersed in the ultrapure water and stored in 11 equal-volume vials, and they were left to stand for 0, 2, 4, 12, 24, 48, 72, 120, 168, 240 and 360 h. And then, the absorbances of the supernatant after magnetic separation were measured. Fig. S5 (c) was the absorbance-time curve.

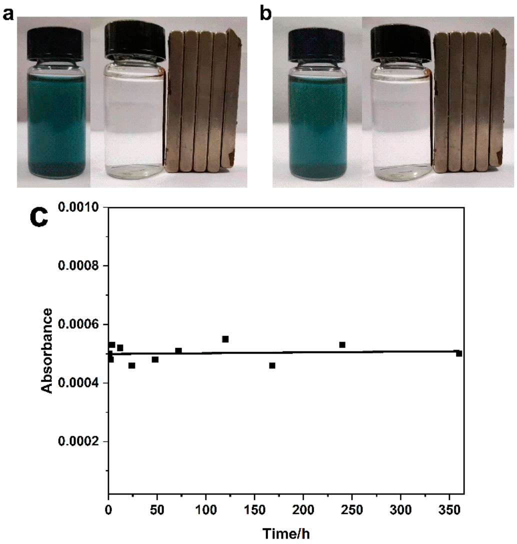

Fig. S5 Stability of liposome composite after standing for different times 1 h(a), 360 h(b), the UV-vis absorbance of MB during 360 hours.

1.Wang S.L., Kong X.Z., Deng Y.J., et al. Neutron Spectrum Measurement with Activation Method in Sample Place of On-line Neutron Activation Analysis System. Nuclear Physics Review 2009,26(1):27-32.

2.Martinho E., Gonçalves I. F., Salgado J. Universal curve of epithermal neutron resonance self-shielding factors in foils, wires and spheres. Applied Radiation and Isotopes 2003,58(3):371-375.
